# Etanercept Suppresses Arteritis in a Murine Model of Kawasaki Disease: A Comparative Study Involving Different Biological Agents

**DOI:** 10.1155/2013/543141

**Published:** 2013-03-31

**Authors:** Ryuji Ohashi, Ryuji Fukazawa, Makoto Watanabe, Hanako Tajima, Noriko Nagi-Miura, Naohito Ohno, Shinichi Tsuchiya, Yuh Fukuda, Shunichi Ogawa, Yasuhiko Itoh

**Affiliations:** ^1^Department of Pathology, Nippon Medical School, 1-1-5 Bunkyo-ku, Sendagi, Tokyo 113-8603, Japan; ^2^Department of Pediatrics, Nippon Medical School, 1-1-5 Bunkyo-ku, Sendagi, Tokyo 113-8603, Japan; ^3^Laboratory for Immunopharmacology of Microbial Products, Tokyo University of Pharmacy and Life Sciences, 1432-1 Horinouchi, Hachioji, Tokyo 192-0355, Japan

## Abstract

Coronary arteritis, a complication of Kawasaki disease (KD), can be refractory to immunoglobulin (IVIG) treatment. To determine the most effective alternative therapy, we compared the efficacy of different agents in a mouse model of KD. Vasculitis was induced by injection of *Candida albicans* water-soluble fractions (CAWS) into a DBA/2 mouse, followed by administration of IVIG, etanercept, methylprednisolone (MP), and cyclosporine-A (CsA). At 2 and 4 weeks, the mice were sacrificed, and plasma cytokines and chemokines were measured. CAWS injection induced active inflammation in the aortic root and coronary arteries. At 2 weeks, the vasculitis was reduced only by etanercept, and this effect persisted for the subsequent 2 weeks. At 4 weeks, IVIG and CsA also attenuated the inflammation, but the effect of etanercept was more significant. MP exerted no apparent effect at 2 or 4 weeks. The suppressive effect exerted by etanercept on cytokines, such as interleukin- (IL-)6, IL-12, IL-13, and tumor necrosis factor-**α** (TNF-**α**), was more evident than that of others. The extent of arteritis correlated with the plasma TNF-**α** levels, suggesting a pivotal role of TNF-**α** in KD. In conclusion, etanercept was most effective in suppressing CAWS-induced vasculitis and can be a new therapeutic intervention for KD.

## 1. Introduction

Kawasaki disease (KD) is an acute, self-limited febrile illness that mainly occurs in early childhood [1]. Most of the clinical symptoms exhibited by patients with KD are caused by an acute inflammatory response affecting small- to medium-sized vessels located throughout the body [1]. The most common complication involves the coronary arteries, leading to prominent vasculitis and secondary aneurysm formation [2]. The injured coronary arteries occasionally cause thrombosis formation, which in turn induces ischemic heart disease [3]. Although the etiology of KD is currently unknown, the condition likely results from an immunologic response triggered by microbial agent [4]. At present, intravenous administration of immunoglobulin (IVIG) is the gold standard therapy for coronary arteritis in the acute phase of KD [5, 6]. However, some patients do not respond to the IVIG therapy, and coronary aneurysms continue to develop in 5% of the affected children [7]. To treat these IVIG nonresponders, alternative treatments, such as corticosteroids, cyclosporine-A, cyclophosphamide, and plasma pheresis have been tested [8–13]. A recent study indicated that IVIG plus prednisolone therapy significantly reduced the occurrence rate of coronary aneurysms in Japanese patients with KD [14]. Despite these clinical trials, the proportion of KD patients with giant coronary aneurysms has remained steady since 2009 [15].

Several cytokines are implicated as significant players in the pathogenesis of KD. Interleukin- (IL-)1, IL-6, IL-10, and tumor necrosis factor- (TNF-)*α*, have been found to be elevated in the serum of KD patients [16–19]. In particular, TNF-*α*, a pleiotropic inflammatory cytokine, has attracted much attention and is known to induce coronary arteritis in KD by upregulating adhesion molecules such as soluble E-selectin [20]. In mice models, anti-TNF-*α* agents have been shown to block the development of a coronary aneurysm [21]. Recently, monoclonal antibodies to TNF-*α* (infliximab) and soluble TNF receptors (etanercept) have emerged as novel therapies for KD; however, their efficacy in suppressing coronary arteritis has not yet been established [22, 23].

Several alternative therapies that may potentially reduce coronary lesions in KD are currently available. However, there has been no comparative analysis on the efficacy of these agents, and the most effective therapy for IVIG-refractory KD remains controversial. In this paper, we tested the efficacy of different agents in a vasculitis mouse model, induced by injection of a *Candida albicans* water-soluble fraction (CAWS). This is a useful animal model of KD because of the many histological similarities observed in this mouse model and KD, including prominent vasculitis in the aortic root and coronary arteries. The degree of inflammatory changes is dependent on the mouse strain; therefore, we chose DBA/2 mice, in which the most severe arteritis can occur [24, 25]. We focused on the therapeutic agents, IVIG, etanercept, methylprednisolone, and cyclosporine-A, in suppressing the progression of inflammation in the heart by comparing histological features and expressions of various plasma cytokines.

## 2. Materials and Methods

The study was performed in accordance with the *Guide for the Care and Use of Laboratory Animals *published by the US National Institute of Health (NIH publication number 85-23, revised 1996). The study protocol was approved by the Animal Care and Use Committee of Nippon Medical School.

### 2.1. Animals

Four-week-old male DBA/2 mice were purchased from Saitama Experimental Animals Supply Co., Ltd. (Saitama, Japan). All mice were maintained under specific pathogen-free conditions, according to the Guidelines for Animals Care of the National Institute of Infectious Diseases in Tokyo (NIID). Water and food were available *ad libitum*. All experimental procedures were approved by the Animal Care and Use Committee of Nippon Medical School.

### 2.2. Preparation of CAWS

CAWS was prepared from *Candida albicans* strain NBRC1358 as previously described [26]. Briefly, 5 L of medium (C-limiting medium) was maintained in a glass incubator for 2 days at 27°C while air was supplied at a rate of 5 L/min and the mixture was swirled at 400 rpm. Following culture, an equal volume of ethanol was added. After allowing the mixture to stand overnight, the precipitate was collected. The precipitate was dissolved in 250 mL of distilled water, and ethanol was added. The mixture was then allowed to stand overnight. The precipitate obtained was collected and dried with acetone to obtain CAWS.

### 2.3. Administration of CAWS and Drugs

CAWS (0.5 mg/mouse/day) in a volume of 0.2 mL was injected into the DBA/2 mouse intraperitoneally on 5 consecutive days from day 0 of the experiment [27]. In a preliminary experiment, we tested regular, double, and triple doses used in the clinical setting of each drug. There was no significant difference in the suppressive effects between the 3 doses. We chose to use the double dose, hypothesizing that we would observe a promising effect in the study (data not shown). Specifically, IVIG (2 g/kg/day) was intraperitoneally injected on 4 consecutive days (IVIG group) [14]. Etanercept (20 mg/kg/day) was subcutaneously injected twice weekly for 2 weeks (EC group) [21]. Methylprednisolone (50 mg/kg/day) was intraperitoneally injected thrice weekly for 3 weeks (MP group) [8]. Cyclosporine-A (10 mg/kg/day) was injected intraperitoneally a day for 2 weeks (CsA group) [11]. For comparison, mice were injected with 0.2 mL of phosphate buffered saline intraperitoneally for 2 weeks after administration of CAWS (CAWS group).

At 2 and 4 weeks of treatment, the mice were sacrificed by an overdose intraperitoneal injection of pentobarbital. Autopsies were performed to obtain plasma and the hearts were fixed in 10% neutralized formalin.

### 2.4. Histological Evaluation

The fixed hearts were embedded in paraffin and sectioned. For detailed observation of the histological changes in the coronary arteries and aorta, 20 to 30 horizontal step sections per mouse were made every 20 *μ*m. Hematoxylin-and-eosin- (H&E-) stained sections were prepared using routine techniques to examine inflammatory changes by light microscopy. In addition, Elastica-Masson Goldner (EMG) staining was performed to further investigate the vascular architecture.

First, we investigated the incidence of panvasculitis in each group. Panvasculitis was defined as the inflammation of all layers of the walls of the coronary arteries and/or the aorta. Second, quantitative evaluation of vascular inflammation was performed as previously described [27]. We divided the areas of the aortic root and coronary arteries into 5 segments (left coronary cusp; right coronary cusp; noncoronary cusp; left coronary artery; and right coronary artery) and graded the intensity of inflammation in each segment as follows: score 3, panvasculitis; score 2, inflammation involving the tunica intima and media but not spreading through the adventitia; score 1, inflammation localized to the tunica intima; and score 0, no inflammatory cell infiltration in the vascular wall. A section with severe inflammation was observed in each segment. The number of segments with at least mild inflammatory changes in each mouse was counted and expressed as mean/group. The severity of inflammation was defined as the sum of the score for the 5 segments. To more accurately quantify the scope of vasculitis, we then measured the total area, including vascular walls and the surrounding connective tissue, infiltrated by inflammatory cells using image analysis software (Image-J, public domain software); this data was expressed as mean (mm^2^) per group.

### 2.5. Measurement of Cytokines and Chemokines

Plasma cytokines and chemokines were measured using a Bio-Plex system. An aliquot of serum (12 *μ*L) was collected from peripheral blood and diluted 4-fold with the dilution solution. The diluted sample was then analyzed for the concentration of cytokines by the 23-Plex kit using Bio-Plex 200 according to the manufacture's protocol and the Bio-Plex Luminex 100 XYP instrument (Bio-Rad, Hercules, CA, USA). We assayed the following 23 cytokines and chemokines: IL-1-alpha(*α*); IL-1-beta(*β*); IL-2; IL-3; IL-4; IL-5; IL-6; IL-9; IL-10; IL-12p40; IL-12p70; IL-13; IL-17; eotaxin; granulocyte-colony stimulating factor (G-CSF); granulocyte macrophage colony-stimulating factor (GM-CSF); *interferon *-*gamma* (IFN-*γ*); keratinocyte-derived *cytokine* (KC); monocyte chemotactic and activating factor (MCP-1); macrophage inflammatory protein-1*α* (MIP-1*α*); MIP-1*β*; regulated upon activation normal T-cell expressed and presumably secreted (RANTES); and TNF-*α*. We used a single assay to a single standard curve provided by the manufacturer. Concentrations of cytokines and chemokines were calculated using the Bio-Plex Manager 3.0 software (Bio-Rad, Tokyo) with a 5-parameter curve-fitting algorithm that is applied for standard curve calculations.

### 2.6. Statistical Analysis

The data on the scope and severity of the arteritis and cytokine/chemokine levels were analyzed using the two-sample *t-*test. The correlation between total area of inflammation and levels of cytokines or chemokines was analyzed using Pearson's test. For all statistical analyses, a value of *P* < 0.05 was considered significant.

## 3. Results

### 3.1. Animal Data

Animal data are summarized in Table 1. At 2 weeks, administration of CAWS and treatment with the drugs had no effect on the body weight (BW), heart weight (HW), and HW/BW ratio (%) in each group.

At 4 weeks, there were no significant changes in BW and HW, but HW/BW ratio of the CAWS group was higher than the control group (*P* < 0.001). There was no increase in HW/BW ratio in all drug-treated groups.

### 3.2. Induction of Vasculitis by CAWS Administration

**Figure 1:**
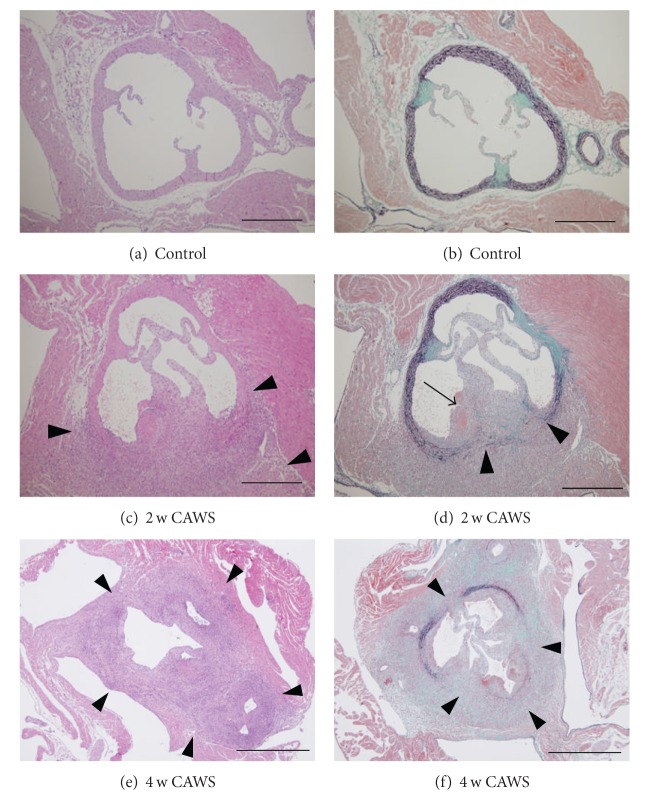
Induction of vasculitis by CAWS administration. Arterial inflammation around the aortic root in control mice (a, b) and the CAWS group (no treatment) at 2 weeks (c, d) and at 4 weeks (e, f). (a) and (b): no significant histological abnormalities are evident in the aortic root and coronary arteries in the negative control group. (c) and (d): at 2 weeks, the active inflammatory infiltrates, composed of numerous neutrophils and lymphocytes, segmentally or partially involved the aortic root ((c), arrowheads). The elastic lamillae are focally disrupted ((d), arrowheads). Exudation of fibrin was seen in the subendothelial region (arrow). (e) and (f): at 4 weeks, the inflammation circumferentially involved the aortic root extending from the adventitia throughout the surrounding connective tissue and heart ventricles ((e), arrowheads). The disruption of the vascular walls due to loss of internal and external elastic lamina was more extensive compared to that at 2 weeks ((f), arrowheads). Chronic changes, represented by accumulation of collagen fibers, progressed. Hematoxylin and eosin (H&E) stain ((a), (c), (e)), Elastica-Masson Goldner (EMG) stain ((b), (d), (f)). (a)–(d) Image at original magnification of ×100; (e) and (f) images at original magnification of ×40. Scale bars: 500 *μ*m (a)–(d) and 1 mm (e, f).

**Figure 2:**
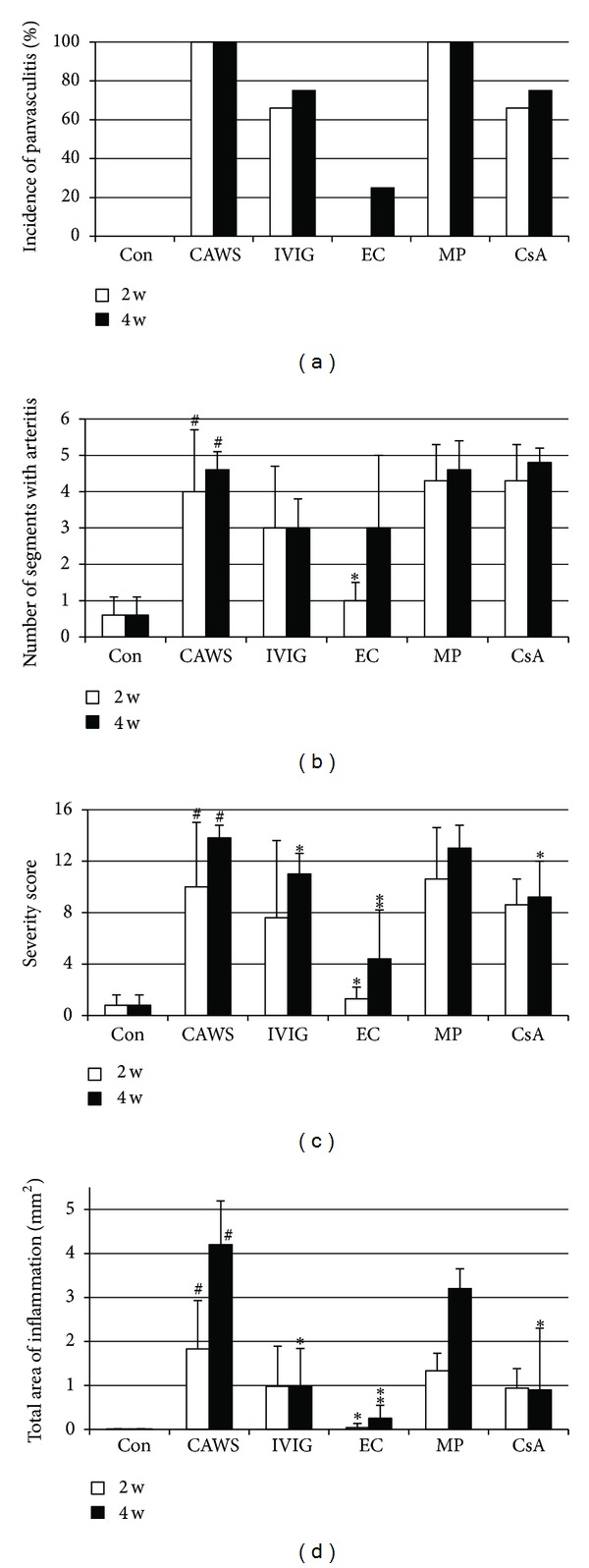
Effects of different biological agents on the development of vasculitis. (a) Incidence of panvasculitis (%), (b) number of segments with arteritis, (c) severity score, and (d) total area of inflammation (mm^2^). Data at 2 weeks are indicated by white bars and those at 4 weeks by black bars. ^#^ *P* < 0.001 versus control, *P < 0.05 versus CAWS, **P < 0.001 versus CAWS. Abbreviations: control (Con), CAWS with no drug treatment (CAWS), immunoglobulin (IVIG), etanercept (EC), methylprednisolone (MP), and cyclosporine-A (CsA).

Two weeks after injection of CAWS, vasculitis developed in the aortic root and coronary arteries in all animals (100%, 3/3) (Figures 1(c), 1(d), and 2(a)). Of 5 aortic segments, 4 ± 1.78 revealed signs of inflammation (Figure 2(b)). The active inflammatory infiltrates, composed of numerous neutrophils and lymphocytes, segmentally or partially involved the aortic root (Figures 1(c), 2(c), and 2(d)). The elastic lamina were focally disrupted with exudation of fibrin, as shown on EMG stain (Figure 1(d)).

At 4 weeks, the panvasculitis progressed in all mice (100%, 5/5) (Figure 2(a)). Of 5 aortic segments, 4.6 ± 0.54 had signs of inflammation (Figure 2(b)), which circumferentially involved the aortic root (Figures 1(e) and 1(f)). In addition to neutrophils, an influx of lymphocytes and histiocytes was evident, resulting in granulomatous inflammation extending from the adventitia to the surrounding connective tissue and heart ventricles. The disruption of the vascular walls due to loss of internal and external elastic lamina was more extensive compared to that at 2 weeks (Figures 1(f), 2(c), and 2(d)). As evidenced by an accumulation of collagen fibers, chronic changes were more prominent, leading to vascular sclerosis. There was a focal finding of endarteritis, resulting in dilatation of the vascular luminal areas, but thrombus formation was not evident within the arterial lumina. 

### 3.3. Effects of Different Biological Agents on Development of Vasculitis

**Figure 3:**
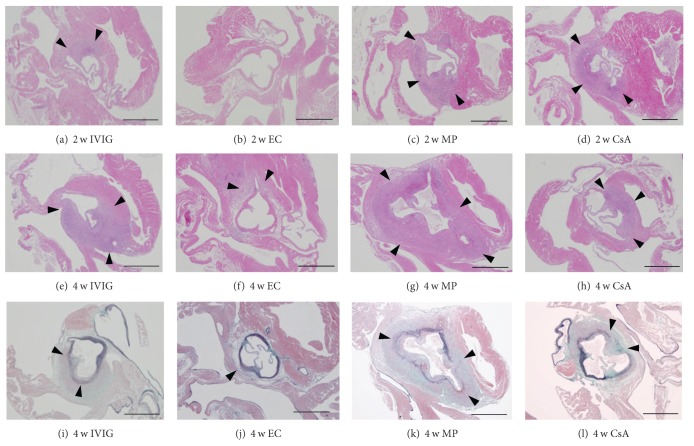
Histological observations on the development of vasculitis treated with different biological agents. Arterial inflammation around the aortic root at 2 weeks (a)–(d) and at 4 weeks (e)–(l) treated with IVIG ((a), (e), (i)), etanercept ((b), (f), (j)), methylprednisolone ((c), (g), (k)), and cyclosporine A ((d), (h), (l)). (a) Segmental inflammatory changes were observed in IVIG-treated mice (arrowheads). (b) Rare significant inflammatory changes were observed yet in etanercept-treated mice. (c, d) In methylprednisolone and cyclosporine-A groups, the inflammatory changes were still segmental (arrowheads), and appeared comparable with each other. (e, i) In IVIG-treated mice at 4 weeks, the inflammatory cell infiltration was more pronounced with loss of elastic lamina (arrowheads) extending into the surrounding tissue. (f, j) In the etanercept group at 4 weeks, vascular architecture was almost preserved except for a small segmental inflammatory area (arrowheads). (g, k) In methylprednisolone-treated mice at 4 weeks, the vascular architecture was diffusely distorted or misshapen due to dense inflammatory cell infiltration that expanded into the hear ventricles and connective tissues (arrowheads). (h, l) In cyclosporine-A-treated mice at 4 weeks, the inflammatory cell infiltration was segmental with focal loss of elastic lamina (arrowheads). H&E stain (a)–(h), EMG stain (i)–(l). All images at original magnification of ×40. All scale bars: 1 mm.

Compared to CAWS group, etanercept treatment after 2 weeks reduced the area of inflammation to a small segment of the vascular walls, decreasing the number of segments with arteritis, the severity score and the total area of inflammation (Figures 2(b), 2(c), and 2(d), *P* < 0.05 versus CAWS, Figure 3(b)). In the IVIG and CsA groups, the extent of inflammation appeared histologically milder than that in the CAWS group at 2 weeks, but was not statistically significant (Figures 2(b), 2(c), 2(d), 3(a), and 3(d)). Injection of methylprednisolone exerted no apparent effects on the development of vasculitis at 2 weeks (Figures 2(b), 2(c), 2(d), and 3(c)).

At 4 weeks, the entire vascular architecture was almost preserved in etanercept-treated mice except for a small segmental inflammatory area (Figures 3(f) and 3(j)). In IVIG and CsA groups, the vasculitis was also attenuated (Figures 3(e), 3(h), 3(i), and 3(l)). However, the decrease in severity score and total area of inflammation was more significant in the EC group compared to IVIG and CsA groups, suggesting that a strong suppressive effect was exerted by etanercept (Figures 2(c) and 2(d), *P* < 0.05 for IVIG and CsA, and *P* < 0.001 for EC). In the MP group, the vascular architecture was diffusely disrupted or lost and replaced by extensive inflammatory infiltrates, similar to that observed in the CAWS group (Figures 2(b), 2(c), 2(d), 3(g), and 3(k)).

### 3.4. Effects of Different Biological Agents on Proinflammatory Cytokine and Chemokine Levels in Plasma

The results of drug efficacy on cytokine levels are summarized in Table 2. At 2 weeks, there was significant elevation of IL-13, IL-17, KC, and TNF-*α* levels in the CAWS group (*P* < 0.001). All the drugs tested suppressed KC expression, and IL-17 was reduced only by etanercept and methylprednisolone. There was no significant reduction in IL-13 and TNF-*α* by any of the drugs.

At 4 weeks, the increased levels of IL-13, IL-17, and TNF-*α* still persisted and were higher than those in the control (CAWS not administered) group (*P* < 0.001). In addition, increased IL-6 and IL-12 levels were also noted after 4 weeks. Etanercept significantly suppressed IL-6, IL-12, IL-13, and TNF-*α* (*P* < 0.001 for IL-6; *P* < 0.05 for IL-12, IL-13, and TNF-*α*). Cyclosporine-A treatment reduced IL-13, IL-17, and TNF-*α* levels (*P* < 0.05). 

### 3.5. Correlation between Plasma Cytokine Levels and Histological Changes

**Figure 4:**
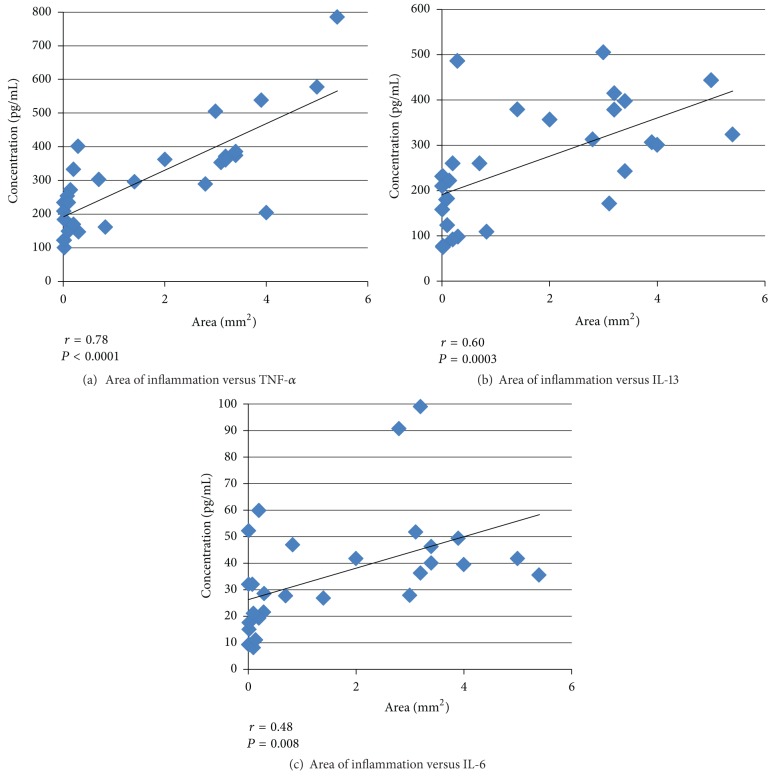
Correlation between histological changes and plasma cytokine levels. Correlation between the total area of inflammation and cytokine levels at 4 weeks. Relationship between the degree of vasculitis, indicated by the total area of inflammation (mm^2^), and plasma levels (pg/mL) of TNF-*α* (a), IL-13 (b), and IL-6 (c).

To evaluate the effects of cytokines on the development of vasculitis, we examined the statistical correlation between the total area of inflammation and levels of each cytokine. At 2 weeks, there was no correlation between the area of inflammation and the level of each cytokine (data not shown). At 4 weeks, the degree of vasculitis, as indicated by the total area of inflammation, correlated with TNF-*α* and IL-13 levels in each animal of all groups (*r* = 0.78, *P* < 0.0001; *r* = 0.60, *P* = 0.0003, resp.) (Figures 4(a), 4(b)). We observed a trend between IL-6 levels and the inflammatory changes (*r* = 0.48, *P* = 0.008) (Figure 4(c)).

## 4. Discussion

Although several drug therapies have been suggested for refractory arteritis in KD, the most effective medication remains controversial. In this study, we compared the efficacy of IVIG, etanercept, methylprednisolone, and cyclosporine-A to treat the vasculitis in DBA/2 mice induced by CAWS injection. To explore the underlying mechanisms, we measured the plasma cytokine levels using a Bio-Plex system. We further examined the relationship between cytokine levels and histological changes. We found that etanercept, an anti-TNF agent, was most effective in attenuating the arteritis by reducing cytokine levels compared to the other drugs studied.

Elevated levels of TNF-*α* have been reported in the sera of patients during the acute phase of KD [19, 28, 29]. In addition to TNF-*α*, several other chemokines and cytokines such as IL-6 and IL-10 are found to be increased in the blood and urine of KD patients [16–19, 30]. In a *Candida albicans*-derived substances (CAD) mice model, another KD vasculitis model, IL-1*β*, IL-6, and TNF-*α* are reported to be upregulated [31]. However, IL-1*α*, IL-13, KC, GM-CSF, MIP-1*α* and TNF-*α* are increased in a CAWS model in C57BL/6N mice [32]. Although patterns of cytokine expression can vary depending on the sample, situation, or animal strain, TNF-*α* is constitutively expressed in all conditions, suggesting a pivotal role of TNF-*α* in KD. In our study, the expression of IL-6, IL-12, IL-13, IL-17, KC, and TNF-*α* was observed at 2 and 4 weeks, but the effect of etanercept treatment was most pronounced, appearing as early as 2 weeks and persisting until 4 weeks. Moreover, the significant correlation between TNF-*α* level and the extent of histological changes suggests a direct link between serum TNF-*α* and inflammatory changes. Thus, our findings confirmed the critical role of TNF-*α* in the development of vasculitis in KD.

TNF-*α*, a well-known proinflammatory cytokine produced by T cells and macrophages, regulates production of chemokines and cytokines and the expression of adhesion molecules on endothelial cells, thereby recruiting circulating leukocytes into the blood flow [33–35]. Therefore, blocking TNF-*α* can be an effective treatment against inflammation. Etanercept and infliximab are 2 currently available pharmacological agents to decrease TNF-*α* activity in KD. Although infliximab is an antibody directed against TNF-*α*, etanercept is a receptor for TNF [36]. Hence, infliximab is more specific for TNF-*α*, while etanercept acts more broadly by binding both TNF-*α* and lymphotoxin. In our study, etanercept treatment not only reduced TNF-*α* levels but also decreased IL-6, IL-12, IL-13, and IL-17. We speculate that by blocking the effect of TNF-*α* or lymphotoxin, etanercept may have reduced other cytokine levels, leading to the attenuation of the vasculitis. Furthermore, we found that the degree of vasculitis correlated most significantly with TNF-*α* levels in each animal in all groups, which suggests that TNF-*α* plays a dominant role in regulating inflammatory changes regardless of the suppressive agents administered. Further studies are necessary to delineate the roles of TNF-*α* in various situations with different types of drugs (not only TNF-*α* suppressive drugs but also other types of anti-inflammatory drugs).

In KD, acute vascular injury involves the coronary arteries and is characterized by acute inflammatory cell infiltration and subsequent aneurysmal formation [2]. A histological study on the coronary arteries from KD patients during the acute phase revealed prominent infiltration of macrophages, and numerous neutrophils in the arterial walls [37]. In animal models induced by CAD or CAWS, similar histological features of vasculitis in the aortic root and coronary arteries have been reported [38, 39]. However, detailed histological analysis is incomplete thus far, as most histological evaluations have been performed by semiquantitative analysis (scoring systems). In our study, we utilized image analysis software to measure the degree of vascular injury in the aortic root and coronary arteries. Thus, we were able to precisely evaluate the extent of inflammatory changes extending from the adventitia into the heart ventricles and surrounding connective tissues. As a result, we could analyze significant differences between the drugs, and we found that anti-TNF-*α* treatment is the most beneficial in attenuating KD vasculitis.

Corticosteroids have typically been used for treating many types of inflammatory diseases and vasculitides. For KD patients, however, the use of steroids has been controversial since the early study by Kato demonstrated that steroids could be detrimental when used as an initial treatment [40]. Nevertheless, a study found that corticosteroid included in an aspirin-containing regimen reduced the occurrence of coronary aneurysm formation in KD [41]. Recent clinical studies have reported that IVIG combined with prednisolone for the initial treatment of acute KD decreased the incidence of coronary artery involvement [14, 42, 43]. In our study, methylprednisolone treatment suppressed the expression of cytokines such as IL-17 and KC at 2 weeks, but eventually failed to exert a significant effect on the evolution of vasculitis. Taken together with the above clinical reports, our study indicates that the effect of steroids may be questionable when used as monotherapy in KD treatment, but could be beneficial when administered with other drugs such as IVIG or aspirin. 

In conclusion, we compared the efficacy of IVIG, etanercept, methylprednisolone, and cyclosporine-A in suppressing the vasculitis occurring in DBA/2 mice induced by the injection of CAWS. We found that the vasculitis was suppressed by IVIG, etanercept, and cyclosporine-A while methylprednisolone failed to show a significant effect. In particular, etanercept was the most effective not only in suppressing inflammation, but also in decreasing plasma cytokine levels. Furthermore, we observed a strong correlation between the extent of the vasculitis and the plasma TNF-*α* levels. Our study suggests that TNF-*α* has an important role in the pathogenesis of vasculitis in KD, and serum levels of TNF-*α* could be a biomarker reflecting the severity of vasculitis of KD. Therefore, etanercept could potentially be a new effective therapy for arteritis in KD. Further clinical trials are required to confirm the efficacy and safety of etanercept therapy and establish its usefulness in the treatment of KD.

## Figures and Tables

**Table 1:** Animal data. Body weight (BW), heart weight (HW), and HW/BW ratio (%) in each group at 2 and 4 weeks.

		Con	CAWS	IVIG	EC	MP	CsA
2 weeks (*n* = 3 for each group)	Body weight (g)	21.7 ± 1.1	20.9 ± 0.1	19.2 ± 0.1	21.3 ± 0.1	19.7 ± 0.1	18.8 ± 0.1
	Heart weight (g)	0.14 ± 0.04	0.13 ± 0.02	0.12 ± 0.02	0.13 ± 0.01	0.12 ± 0.01	0.12 ± 0.01
	HW/BW (%)	0.62 ± 0.1	0.60 ± 0.06	0.60 ± 0.05	0.61 ± 0.04	0.59 ± 0.01	0.62 ± 0.08

4 weeks (*n* = 5 for each group)	Body weight (g)	22.2 ± 0.14	20.9 ± 0.2	22.8 ± 0.1	21.4 ± 0.1	19.5 ± 0.1	22.4 ± 0.1
	Heart weight (g)	0.14 ± 0.01	0.18 ± 0.02	0.14 ± 0.02	0.15 ± 0.02	0.13 ± 0.01	0.13 ± 0.03
	HW/BW (%)	0.63 ± 0.06	0.86 ± 0.07^#^	0.59 ± 0.07	0.68 ± 0.07	0.68 ± 0.05	0.57 ± 0.14

^#^ *P* < 0.001 versus Con.

Abbreviations: control (Con), CAWS with no drug treatment (CAWS), immunoglobulin (IVIG), etanercept (EC), methylprednisolone (MP), and cyclosporine-A (CsA).

**Table 2:** Proinflammatory cytokine levels in plasma. At 2 weeks, plasma levels of IL-13, IL-17, KC, and TNF-*α* were increased in the CAWS group, compared to negative control group. KC was suppressed by all the drugs tested, and IL-17 was reduced by etanercept and methylprednisolone treatment. At 4 weeks, the increased levels of IL-13, IL-17, and TNF-*α* persisted in the CAWS group with an increase in IL-6 and IL-12 levels. Etanercept significantly suppressed the levels of IL-6, IL-12, IL-13, and TNF-*α*, whereas cyclosporine-A reduced IL-13, IL-17, and TNF-*α* levels.

		Con	CAWS	IVIG	EC	MP	CsA
	IL-13	142 ± 69.2	277 ± 98.8^#^	423 ± 333	376 ± 361	214 ± 97.1	256 ± 138
2 weeks	IL-17	7.71 ± 3.78	44 ± 17.7^#^	22.5 ± 14.5	11.3 ± 9.23*	8.03 ± 4.62*	12.6 ± 2.88
	KC	24.4 ± 31.4	113 ± 61.5^#^	4.83 ± 1.49*	9.54 ± 6.68*	7.47 ± 0.78*	9.77 ± 5.14*
	TNF-*α*	152 ± 54.9	276 ± 75.1^#^	253 ± 251	217 ± 49.3	205 ± 118	227 ± 163

	IL-6	20.1 ± 18.6	43.7 ± 6.72^#^	29.7 ± 8.6*	16.8 ± 7.63**	66.5 ± 46.9	41.2 ± 14.9
	IL-12	353 ± 155	577 ± 167^#^	458 ± 157	253 ± 104*	253 ± 70*	495 ± 298
4 weeks	IL-13	170 ± 64.6	328 ± 103^#^	330 ± 164	188 ± 84.3*	382 ± 82.9	161 ± 61*
	IL-17	9.7 ± 4.07	34.5 ± 10.2^#^	28.3 ± 19.3	26 ± 18.2	61.9 ± 36.2	11.1 ± 8.76*
	TNF-*α*	166 ± 56.7	525 ± 175^#^	301 ± 112	247 ± 78.2*	346 ± 111	240 ± 90*

^#^ *P* < 0.001 versus Con.

*P < 0.05 versus CAWS.

**P < 0.001 versus CAWS.
